# Rice ETHYLENE RESPONSE FACTOR 101 Promotes Leaf Senescence Through Jasmonic Acid-Mediated Regulation of *OsNAP* and *OsMYC2*

**DOI:** 10.3389/fpls.2020.01096

**Published:** 2020-07-16

**Authors:** Chaemyeong Lim, Kiyoon Kang, Yejin Shim, Yasuhito Sakuraba, Gynheung An, Nam-Chon Paek

**Affiliations:** ^1^Department of Plant Science, Plant Genomics and Breeding Institute, Research Institute of Agriculture and Life Sciences, Seoul National University, Seoul, South Korea; ^2^Division of Life Sciences, Incheon National University, Incheon, South Korea; ^3^Graduate School of Agricultural and Life Sciences, Biotechnology Research Center, The University of Tokyo, Tokyo, Japan; ^4^Department of Plant Molecular Systems Biotechnology, Crop Biotech Institute, Kyung Hee University, Yongin, South Korea

**Keywords:** rice, leaf senescence, chlorophyll degradation, jasmonic acid signaling, *OsERF101*, *OsNAP*, *OsMYC2*

## Abstract

Leaf senescence is the final stage of leaf development and an important step that relocates nutrients for grain filling in cereal crops. Senescence occurs in an age-dependent manner and under unfavorable environmental conditions such as deep shade, water deficit, and high salinity stresses. Although many transcription factors that modulate leaf senescence have been identified, the mechanisms that regulate leaf senescence in response to environmental conditions remain elusive. Here, we show that rice (*Oryza sativa*) *ETHYLENE RESPONSE FACTOR 101* (*OsERF101*) promotes the onset and progression of leaf senescence. *OsERF101* encodes a predicted transcription factor and *OsERF101* transcript levels rapidly increased in rice leaves during dark-induced senescence (DIS), indicating that OsERF101 is a senescence-associated transcription factor. Compared with wild type, the *oserf101* T-DNA knockout mutant showed delayed leaf yellowing and higher chlorophyll contents during DIS and natural senescence. Consistent with its delayed-yellowing phenotype, the *oserf101* mutant exhibited downregulation of genes involved in chlorophyll degradation, including rice *NAM*, *ATAF1/2*, and *CUC2* (*OsNAP*), *STAY-GREEN* (*SGR*), *NON-YELLOW COLORING 1* (*NYC1*), and *NYC3* during DIS. After methyl jasmonate treatment to induce rapid leaf de-greening, the *oserf101* leaves retained more chlorophyll compared with wild type, indicating that *OsERF101* is involved in promoting jasmonic acid (JA)-induced leaf senescence. Consistent with the involvement of JA, the expression of the JA signaling genes *OsMYC2/JA INSENSITIVE 1* (*OsJAI1*) and *CORONATINE INSENSITIVE 1a* (*OsCOI1a*), was downregulated in the *oserf101* leaves during DIS. Transient transactivation and chromatin immunoprecipitation assays revealed that OsERF101 directly binds to the promoter regions of *OsNAP* and *OsMYC2*, which activate genes involved in chlorophyll degradation and JA signaling-mediated leaf senescence. These results demonstrate that *OsERF101* promotes the onset and progression of leaf senescence through a JA-mediated signaling pathway.

## Introduction

Leaf senescence is the final stage of leaf development and involves many molecular and physiological events, including chlorophyll degradation, the breakdown of other cellular components, and cell death, thus allowing plants to recycle nutrients from leaf tissues into seeds (Masclaux-Daubresse et al., 2008; Distelfeld et al., 2014; Yang et al., 2019). Environmental stresses, such as deep shade, water deficit, extreme temperatures, and high salinity, accelerate leaf senescence (Munné-Bosch and Alegre, 2004; Mickelbart et al., 2015). Premature leaf senescence shortens the vegetative growth period, inducing the precocious transition from the vegetative to the reproductive stage and reducing the plant’s nutritional capacity, thus negatively affecting crop productivity.

Age-dependent and stress-induced senescence share many regulatory networks and physiological, biochemical, and molecular mechanisms (Gepstein and Glick, 2013; Zhuang et al., 2019). Transcription factors (TFs) participate in the regulatory pathways for leaf senescence and abiotic stress tolerance. For instance, RNA interference (RNAi)-mediated suppression of rice (*Oryza sativa*) *NAC2* (*OsNAC2*), a member of the NAC family, reduces the expression of chlorophyll-degradation and senescence-associated genes, leading to delayed leaf yellowing during dark-induced senescence (DIS) (Mao et al., 2017). Moreover, *OsNAC2*-RNAi transgenic plants display enhanced tolerance against drought and high salt stresses due to upregulation of stress-related and abscisic acid (ABA)-signaling genes (Shen et al., 2017). Overexpression of *ONAC106* causes delayed leaf yellowing in rice, resulting in extended photosynthetic capacity at the reproductive stage in paddy fields and increased grain production. In addition to the biological function of *ONAC106* in leaf senescence, transgenic rice overexpressing *ONAC106* exhibit improved tolerance to salt stress (Sakuraba et al., 2015). The gain-of-function mutant *prematurely senile 1-D* (*ps1-D*) exhibits upregulated expression of *OsNAP*, leading to accelerated leaf yellowing during both natural senescence and DIS (Liang et al., 2014). In addition, overexpression of *OsNAP* confers tolerance to salt and drought stresses (Chen et al., 2014). These distinct phenomena reveal the role of *OsNAP* in regulating diverse genes involved in chlorophyll degradation and abiotic stress responses.

Plant hormones serve as signal molecules and are involved in the regulation of a variety of cellular processes, including leaf senescence and abiotic stress responses. Among them, jasmonic acid (JA) accumulates to high levels in plant cells when plants enter the senescence phase or in unfavorable conditions (He et al., 2002; Wasternack and Hause, 2013; Hou et al., 2016), JA biosynthesis is catalyzed by enzymes including lipoxygenase (LOX), allene oxide synthase (AOS), allene oxide cyclase (AOC), and 12-oxo-PDA reductase (OPR) (He et al., 2002). Increased JA levels activate the downstream signal transduction pathways that are involved in senescence and abiotic stress tolerance. For instance, elevated JA levels are perceived by the F-box protein CORONATINE INSENSITIVE 1 (COI1), followed by ubiquitination and degradation of JA ZIM-domain (JAZ) proteins (Niu et al., 2011; Song et al., 2011; Qi et al., 2014). JAZ degradation causes the release of downstream TFs such as the basic helix-loop-helix (bHLH) TF family members MYC2, MYC3, and MYC4 (Fernández-Calvo et al., 2011). The *oscoi1b* mutant leaves retain their green color during DIS (Lee et al., 2015). Transgenic plants overexpressing a truncated OsJAZ8 protein that which lacks the ubiquitin-binding Jas domain also exhibit a delayed senescence phenotype (Uji et al., 2017). OsMYC2/OsJAI1 directly binds to the promoter of senescence-associated genes, and thus its overexpression leads to leaf yellowing during DIS (Uji et al., 2017). In Arabidopsis, MYC2/JAI1 and its homologous proteins MYC3 and MYC4 regulate the expression of the chlorophyll degradation genes *NON-YELLOWING 1* (*NYE1*)*, NON-YELLOW COLORING 1* (*NYC1*), and *PHEOPHYTINASE* (*PPH*) by binding to their promoters (Zhu et al., 2015).

The APETALA2/Ethylene Response Factor (AP2/ERF) family can be divided into four subfamilies depending on the number of AP2/ERF domains and their amino acid similarity, such as APETALA2 (AP2), Related to ABI3/VP1 (RAV), Dehydration Responsive Element Binding protein (DREB), and Ethylene Response Factor (ERF) subfamilies (Sakuma et al., 2002; Nakano et al., 2006). DREB and ERF subfamilies have a single AP2/ERF domain that can bind to both the C-Repeat/Dehydration Responsive Element (CRT/DRE) and the Ethylene Response Element (ERE) in the promoter regions of their target genes. ERFs regulate the expression of abiotic stress-related genes in various plant species including rice (Fukao et al., 2011), Arabidopsis (Phukan et al., 2017), wheat (*Triticum aestivum*) (Gao et al., 2018), maize (*Zea mays*) (Liu et al., 2013), soybean (*Glycine max*) (Zhang et al., 2009), and tobacco (*Nicotiana tabacum*) (Park et al., 2001).

A recent study showed that the rice *ETHYLENE RESPONSE FACTOR 101* (*OsERF101*) plays an important role in enhancing tolerance to drought stress in reproductive tissues (Jin et al., 2018). However, the regulatory functions of *OsERF101* in leaf senescence have not been understood. In this study, our results substantially showed that OsERF101 positively regulates leaf senescence in rice, and directly activates the expression of *OsNAP* and *OsMYC2* that play a central role in mediating chlorophyll degradation and JA-mediated leaf senescence. Thus, our findings provide a new molecular insight of *OsERF101* function in leaf senescence.

## Materials and Methods

### Plant Materials, Growth Conditions, and Dark, Phytohormone, and Stress Treatments

The T-DNA insertion mutant *oserf101* (PFG_2D-00368) was obtained from Crop Biotech Institute at Kyung Hee University, Republic of Korea (Jeong et al., 2002). The *Oryza sativa japonica* cultivar ’Dongjin’ (parental line) and the *oserf101* mutant were cultivated in a paddy field under natural long day (NLD) conditions (>14 h sunlight/day, 37°N latitude) in Suwon, Republic of Korea. The germinated rice seedlings were transplanted in a paddy soil and grown in a growth chamber under long day (LD) conditions (14 h light/10 h dark, 37°N latitude) in Seoul, Republic of Korea. For the dark treatment, the detached leaves of rice plants grown in a growth chamber under LD conditions for 3 weeks were incubated on 3 mM MES (pH 5.8) buffer at 28°C in complete darkness. For phytohormone and stress treatments, the sterilized seeds were germinated on half-strength Murashige and Skoog (1/2 MS) solid medium under continuous light (90 µmol m^-2^ s^-1^) at 30°C. The 10-day-old seedlings were transferred to 1/2 MS liquid medium containing 100 µM 1-aminocyclo-propane-1-carboxylic acid (ACC), 100 µM MeJA, 100 µM ABA, 100 mM NaCl, and 20% polyethylene glycol (PEG) or were dehydrated by air drying. Rice seedlings incubated in 1/2 MS liquid medium without additional phytohormones were used as a mock control.

### Determination of Total Chlorophyll and Photosynthetic Capacity

To measure the total chlorophyll, pigments were extracted from an equal fresh weight of rice leaves grown in a paddy field, incubated in complete darkness, or treated with 100 µM MeJA using 80% ice-cold acetone. The extracts were centrifuged at 10,000 g for 10 min at 10°C and then the absorbance of the supernatants was measured at 647 and 663 nm using an UV/VIS spectrophotometer (BioTek). The concentration of chlorophyll was calculated as previously described (Porra et al., 1989). To determine the photosynthetic activity, the *Fv/Fm* ratio was measured using the OS-30p+ instrument (Opti-Sciences). The middle part of each flag leaf of plants grown in a paddy field under NLD conditions was adapted in the dark for 5 min and then the *Fv/Fm* ratio was determined in the flag leaves.

### Determination of Phytohormone Sensitivity

To determine the sensitivity to phytohormones, detached leaves of 3-week-old plants grown in paddy soil were floated on 3 mM MES (pH 5.8) buffer containing 50 µM MeJA and 50 µM ABA and incubated in continuous light conditions (90 µmole·m^−2^s^−1^) at 30°C for 4 days. Detached leaves floated on 3 mM MES buffer (pH 5.8) without phytohormones were used as a control. To assess the ABA sensitivity of rice seedlings, the 10-day-old rice seedlings grown in 1/2 MS solid medium were transferred to 1/2 MS liquid medium containing 5 and 10 µM ABA. Seedlings incubated in 1/2 MS liquid medium without additional phytohormones were used as a mock control.

### Quantitative Reverse Transcription PCR (qRT-PCR) and Semiquantitative RT-PCR

Total RNA was extracted from rice tissues using an RNA Extraction Kit (Macrogen, South Korea) according to manufacturer instructions. First-strand cDNA was synthesized with 2 µg of total RNA in a 25 µl volume using M-MLV reverse transcriptase and oligo(dT)_15_ primer (Promega), and diluted with 75 µl of water. The qPCR amplifications were conducted on the LightCycler 2.0 instrument (Roche Diagnostics). The 20 µl of qPCR mixture included 2 µl of the first-strand cDNA mixture, 10 µl of 2X GoTaq PCR Master Mix (Promega), and 1 µl of 10 pM primer pairs (**Supplementary Table S1**). The qPCR conditions were 95°C for 2 min, followed by 50 cycles at 95°C for 5 s, 59°C for 15 s, and 72°C for 10 s. The rice *UBIQUITIN5* (*OsUBQ5*, AK061988) gene was used as an internal control for normalization. The semi-quantitative PCR was performed in a 20 µl volume containing 2 µl diluted cDNA, 1 unit Ex Taq polymerase (TaKaRa Biotechniques), and 1 µl of 10 pM primers (listed in **Supplementary Table S1**). The PCR program included initial denaturation at 94°C for 3 min, followed by specified cycles at 94°C for 30 sec, 55°C for 1 min, and 72°C for 40 sec, followed by a final extension at 72°C for 5 min. The PCR products were electrophoresed on a 1% agarose gel. *OsUBQ5* was used as an equal loading control.

### Plasmid Construction and Rice Transformation

To generate the overexpression construct, a full-length cDNA of *OsERF101* was amplified using the primers listed in **Supplementary Table S1**. The amplified fragments were ligated into the pCR8/GW/TOPO TA cloning vector (Invitrogen) and then inserted into the pMDC32 Gateway-compatible binary vector through the LR recombination reaction. The pMDC32-OsERF101 plasmids were introduced into callus of Dongjin seeds by *Agrobacterium tumefaciens* (strain LBA4404)-mediated transformation (Jeon et al., 2000). Agrobacterium-infected calli were transferred to 1/2 MS solid medium containing cytokinin and auxin. Plantlets regenerated from the callus were grown under continuous light conditions (90 µmole·m^−2^ s^−1^) at 30°C.

### Protoplast Transient Transactivation Assays

Reporter plasmids were constructed by the insertion of *OsMYC2* (-1529 to -1 bp) and *OsNAP* (-1502 to -1 bp) promoters into the pJD301 vector (Luehrsen et al., 1992). To construct effector plasmids, *OsERF101* cDNA from the WT cultivar was cloned upstream of a sequence encoding six copies of a MYC epitope tag in the pGA3697 vector. Protoplasts were isolated from young rice seedlings as previously described (Zhang et al., 2011). A reporter plasmid (4 µg) and an effector plasmid (8 µg) were co-transfected, together with 1 µg of an internal control plasmid (pUBQ10-GUS), into 5 × 10^4^ protoplasts using the polyethylene glycol (PEG)-mediated transfection method (Yoo et al., 2007). Transfected protoplasts were incubated in protoplast culture solution (0.4 M mannitol, 15 mM MgCl_2_, 4 mM MES-KOH [pH 5.8]) in the dark at room temperature for 16 h. Luciferase (LUC) activity in each cell lysate was determined using the Luciferase Assay System Kit (Promega). LUC activity was normalized against β-glucuronidase (GUS) activity derived from the internal control plasmid.

### Chromatin Immunoprecipitation (ChIP) Assays

For the ChIP assay, the *Ubi : OsERF101-Myc* and *Ubi : Myc* were transfected into rice protoplasts as previously described (Zhang et al., 2011). Protoplasts were then subjected to cross-linking with 1% formaldehyde for 30 min under vacuum. Then, nuclei were isolated and lysed, and chromatin complexes were isolated and sonicated, as described (Saleh et al., 2008). DNA was sonicated using a BIORUPTORII (COSMO BIO). Anti-Myc monoclonal antibody (Abcam, Cambridge, UK) and protein A agarose beads (Merck Millipore) were used for immunoprecipitation. DNA recovered from agarose beads was purified using the DNeasy Plant Mini Kit (Qiagen). Quantitative PCR was performed using the KAPA SYBR FAST qPCR Kit (KAPA Biosystems) and gene-specific primers (**Supplementary Table S1**).

## Results

### Expression of *OsERF101* in Rice

Rice *OsERF101* (LOC_Os04g32620) consists of 4366 bp of genomic DNA with an 807-bp open reading frame containing two exons and encoding a 268-amino acid protein belonging to the ERF transcription factor subfamily. Amino acid sequence alignments between OsERF101 and its putative orthologs demonstrated that they have a single AP2/ERF domain, which is highly conserved in plants (**Supplementary Figure S1**).

**Figure 1:**
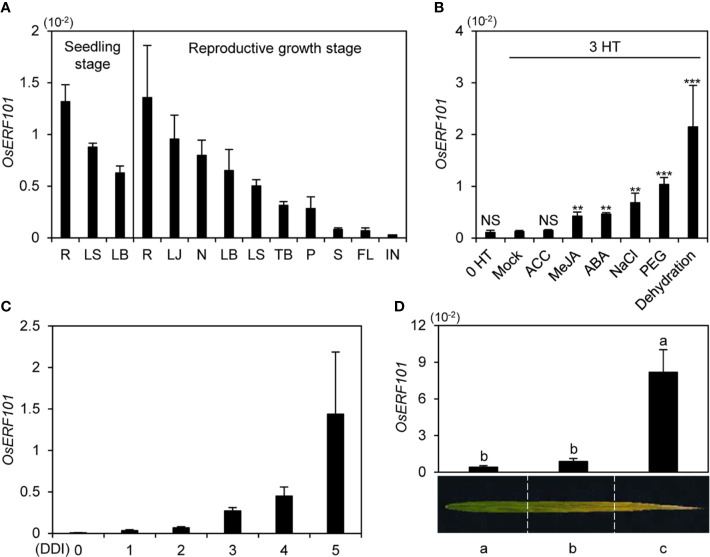
Expression profiles of *OsERF101*. **(A)** *OsERF101* was differentially expressed in various *japonica* cultivar ’Dongjin’ (hereafter wild type; WT) tissues: root (R), leaf sheath (LS), leaf blade (LB), lamina joint (LJ), node (N), tiller base (TB), panicle (P), stem (S), flag leaf (FL), and internode (IN). WT seedlings were grown in a growth chamber for 3 weeks under long-day (LD) conditions (14 h light/10 h dark). WT plants were cultivated in a paddy field until the reproductive stage at 120 days after seeding (DAS) under natural long-day (NLD) conditions (>14 h light/day). **(B)** Expression patterns of *OsERF101* in response to abiotic stresses and phytohormones. WT seedlings grown in half-strength Murashige and Skoog (1/2 MS) solid medium for 10 d under continuous light at 28°C were incubated in 1/2 MS liquid medium supplemented with 100 µM ACC, 100 µM MeJA, 100 µM ABA, 100 mM NaCl, or 20% PEG or dehydrated by air drying. Seedlings incubated in 1/2 MS liquid medium without treatment were used as a mock control. Total RNA was isolated from the leaves at 0 and 3 h of treatment (HT). Asterisks indicate statistically significant differences between treated samples and the mock control, as determined by Student’s t-test (**p < 0.01, ***p < 0.001). NS, not significant. **(C)** *OsERF101* expression gradually increased in detached leaves of 3-week-old WT plants grown in a growth chamber under LD conditions. Detached leaves were subjected to complete darkness in 3 mM MES (pH 5.8) at 28°C. **(D)** Expression of *OsERF101* was measured in flag leaves divided into three regions from the green sector (a) to the yellow sector (c) at the ripening stage (130 DAS). *OsERF101* mRNA levels were determined by qRT-PCR analysis and normalized to that of *OsUBQ5* (Os01g22490). Mean and standard deviations were obtained from at least three biological samples. Different letters indicate significantly different values according to a one-way ANOVA and Duncan’s least significant range test (*p* < 0.05). Experiments were repeated twice with similar results.

To investigate the expression of *OsERF101*, we measured *OsERF101* transcript levels in various organs (root, leaf sheath, leaf blade, lamina joint, node, tiller base, panicle, stem, flag leaf, and internode). We used wild-type (WT; *japonica* rice cultivar ‘Dongjin’) plants grown in a growth chamber under long-day (LD) conditions (14.5 h light/9.5 h dark) at 21 days after germination (DAG, seedling stage) or in the paddy field under natural LD (NLD) conditions (≥14 h light/day in Suwon, South Korea, 37°N latitude) at 120 DAG (heading stage). Reverse transcription and quantitative real-time PCR (qRT-PCR) analysis revealed that the *OsERF101* transcripts were most abundant in root, and then (in order of decreasing abundance), in lamina joint, node, leaf blade, and leaf sheath (**Figure 1A**).

To test whether phytohormones and stress treatments affect the expression of *OsERF101*, we examined the *OsERF101* transcript levels in 10-day-old WT seedlings treated with 1-aminocyclo-propane-1-carboxylic acid (ACC), MeJA, ABA, NaCl, PEG, or under dehydration stress for 3 h. qRT-PCR analysis showed that *OsERF101* expression increased significantly in response to the MeJA, ABA, NaCl, PEG, and water deficit treatments (**Figure 1B**). In addition, *OsERF101* expression was sharply upregulated in the detached leaves of 2-week-old seedlings during DIS (**Figure 1C**). In the naturally senescing flag leaves, *OsERF101* transcripts accumulated to higher levels in the yellowed tip (**c**) region than in the yellowing middle (**b**) or green bottom (**a**) regions (**Figure 1D**). These results strongly suggested that *OsERF101* is involved in leaf senescence in rice.

### *OsERF101* Positively Regulates the Onset and Progression of Leaf Senescence

**Figure 2:**
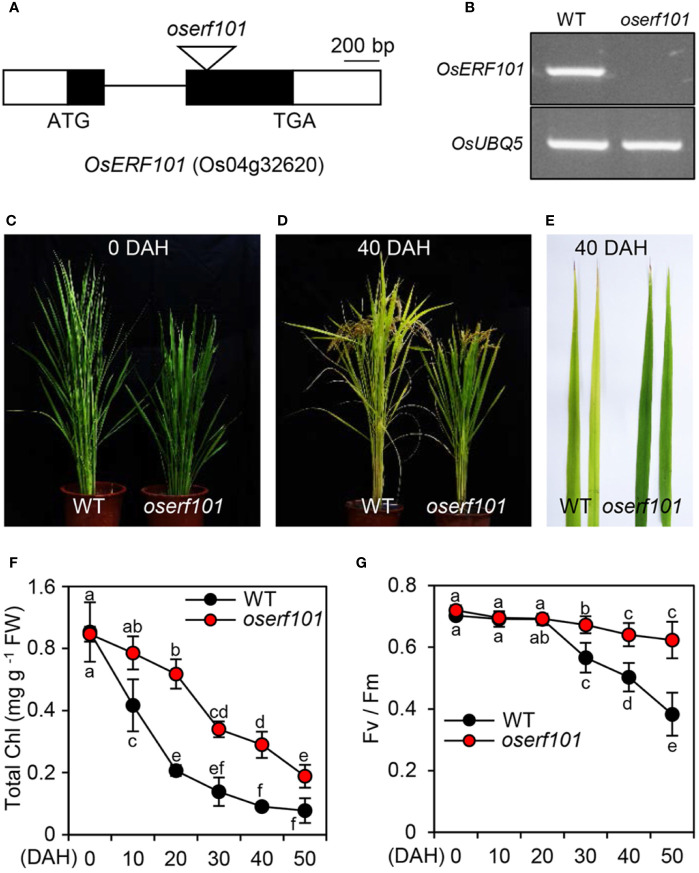
The *oserf101* mutant exhibits delayed natural leaf senescence. **(A)** Position of the T-DNA insertion in the exon region of *OsERF101* (LOC_Os04g32620). Black and white boxes represent exons and untranslated regions, respectively. The black line indicates an intron and the open triangle indicates the location of the T-DNA insertion (*oserf101*, PFG_2D-00368). **(B)** Mutation of *OsERF101* was confirmed by semi-quantitative RT-PCR. Total RNA was isolated from detached second leaves of a 3-week-old WT (the parental *japonica* rice cultivar ’Dongjin’) plant and the *oserf101* mutant in which senescence was induced for 3 days of dark incubation as shown in Figure 3A. *OsUBQ5* (Os01g22490) was used as a loading control. **(C, D)** The comparison of senescence phenotypes of WT and *oserf101* plants at 0 and 40 days after heading (DAH) under natural long-day conditions (>14 h light/day). **(E)** Senescing flag leaves of WT and *oserf101* plants at 50 DAH. The images are representative of five independent experiments. **(F, G)** Changes in total chlorophyll (Chl) contents **(F)** and photosynthetic capacity (Fv/Fm) **(G)** of WT and *oserf101* flag leaves after heading. Mean and standard deviations were obtained from more than 10 plants. Different letters indicate significantly different values according to a one-way ANOVA and Duncan’s least significant range test (*p* < 0.05).

To examine the role of the OsERF101 TF in regulating leaf senescence, we obtained a loss-of-function mutant (PFG_2D-00368) from RiceGE (://signal.salk.edu/cgi-bin/RiceGE), in which a T-DNA fragment was integrated into the second exon of *OsERF101* (**Figure 2A**). To verify the effect of the T-DNA insertion, we performed semi-quantitative RT-PCR analysis and found that in the detached leaves of 3-week-old plants, the *OsERF101* transcript was completely absent in the PFG_2D-00368 line (**Figure 2B**) at 3 days of dark incubation (DDI), indicating that the PFG_2D-00368 line is a knockout mutant (hereafter termed *oserf101*). We next examined the progress of senescence in WT and *oserf101* plants grown in the paddy field under NLD conditions. While there was no significant difference in leaf color between WT and *oserf101* plants at 0 days after heading (DAH) (**Figure 2C**), the *oserf101* leaves retained their green color during grain filling much more than the WT (**Figures 2D, E**). Consistent with the persistence of green leaf color, the chlorophyll levels remained much higher in the flag leaves of the *oserf101* mutant compared to the WT (**Figure 2F**). In addition, the *oserf101* mutant maintained a higher *Fv/Fm* ratio (efficiency of photosystem II) compared to the WT after 20 DAH (**Figure 2G**), indicating that the prolonged greenness of *oserf101* leaves leads to improved photosynthetic activity during grain filling in the autumn fields.

**Figure 3:**
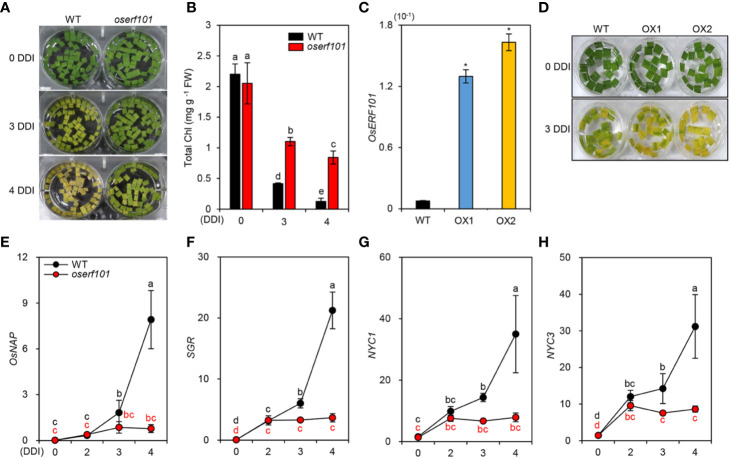
The *oserf101* mutant exhibited delayed leaf yellowing during dark-induced senescence (DIS) conditions. **(A, B)** Detached leaves of 3-week-old WT and *oserf101* plants grown in the greenhouse under natural long-day conditions (>14 h light/day) were incubated in 3 mM MES buffer (pH 5.8) with the abaxial side up at 28°C under complete darkness. The visual phenotypes **(A)** and total chlorophyll (Chl) contents **(B)** were observed after 0, 3, and 4 days of dark incubation (DDI). Different letters indicate significantly different values according to a one-way ANOVA and Duncan’s least significant range test (*p* < 0.05). **(C)** Expression of *OsERF101* measured in the leaves of WT and *OsERF101*-overexpressing (*OsERF101*-OX) transgenic plants (OX1, OX2) grown in the greenhouse for one month. **(D)** Detached leaves of 1-month-old *OsERF101*-OX plants exhibited accelerated leaf yellowing at 3 DDI. **(E–H)** Altered expression of *OsNAP* and chlorophyll degradation genes in detached leaves of 3-week-old WT and *oserf101* seedlings during DIS. Total RNA was isolated from detached leaves at 0, 2, 3, and 4 DDI. The transcript levels of *OsERF101* **(C)**, *OsNAP* **(E)**, and chlorophyll degradation genes **(F–H)** were determined by qRT-PCR and normalized to that of *OsUBQ5* (Os01g22490). **(C)** Asterisks indicate statistically significant differences between WT and *OsERF101*-OX plants, as determined by Student’s t-test (*p < 0.05). **(E–H)** Different letters indicate significantly different values according to a one-way ANOVA and Duncan’s least significant range test (*p* < 0.05). Mean and standard deviations were obtained from at least three biological samples. Experiments were repeated twice with similar results. FW, fresh weight.

To further examine the effects of *OsERF101* on leaf de-greening in DIS, we monitored the color in sections of leaves detached from 3-week-old WT and *oserf101* plants grown under LD conditions. The *oserf101* leaves retained their green color much longer than the WT leaves (**Figure 3A**), consistent with their relatively higher chlorophyll contents at 3 and 4 DDI (**Figure 3B**). To confirm the positive role of *OsERF101* in leaf senescence, we generated two independent transgenic rice plants overexpressing *OsERF101* (*OsERF101*-OX1 and -OX2) (**Figure 3C**). The detached leaves of 3-week-old *OsERF101*-OX plants exhibited accelerated leaf yellowing compared to those of the WT at 3 DDI (**Figure 3D**). These results indicate that OsERF101 is a positive regulator of the onset and progression of leaf senescence.

Among the rice regulatory genes controlling leaf senescence, *OsNAP* expression is induced by senescence, and OsNAP directly activates the transcription of chlorophyll degradation genes (Liang et al., 2014) including *STAY-GREEN* (*SGR*, Park et al., 2007), *NYC1* (Kusaba et al., 2007), and *NYC3* (Morita et al., 2009). To investigate whether *OsERF101* affects the expression of *OsNAP* and chlorophyll degradation genes, we measured their transcript levels in the detached leaves of 3-week-old WT and *oserf101* seedlings during DIS. The qRT-PCR results showed that, while the transcript levels of *OsNAP* and chlorophyll degradation genes increased in the WT at 3 DDI, their expression was not altered in the *oserf101* mutant during DIS (**Figures 3E–G**). These results indicate that *OsERF101* acts as a positive regulator of leaf senescence by upregulating the expression of *OsNAP* and chlorophyll degradation genes.

### OsERF101 Acts in JA-Mediated Leaf Senescence

**Figure 4:**
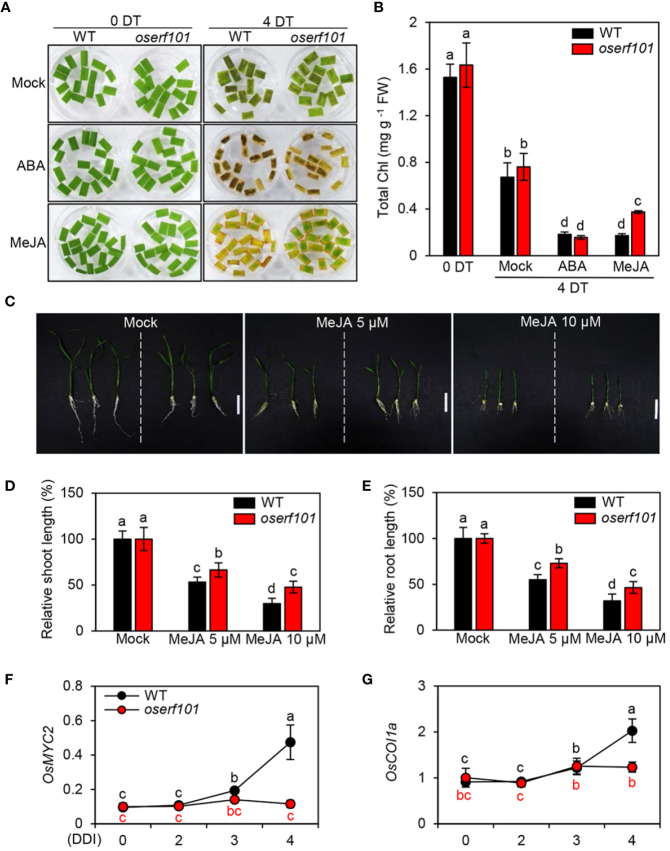
Jasmonic acid (JA) hyposensitivity of the *oserf101* mutants. **(A, B)** Detached leaves of 3-week-old WT and os*erf101* plants grown in the greenhouse under natural long-day conditions (>14 h light/day) were incubated in 3 mM MES buffer (pH 5.8) containing 50 µM abscisic acid (ABA) or 50 µM methyl jasmonate (MeJA) under continuous light at 28°C. Detached leaves floated on 3 mM MES buffer (pH 5.8) without phytohormones were used as a mock control. The MeJA hyposensitive phenotype **(A)** and total chlorophyll (Chl) contents **(B)** were observed at 0 and 4 days after treatment (DT). **(C–E)** The WT and *oserf101* seeds germinated in half-strength Murashige and Skoog (1/2 MS) solid medium for 3 d were grown in 1/2 MS solid medium containing 5 or 10 µM MeJA for 10 d under long-day (LD) conditions (14 h light at 30°C/10 h dark at 25°C). Seedlings grown in 1/2 MS solid medium without MeJA were used as a mock control. The growth phenotype **(C)** and relative shoot **(D)** and root **(E)** length were observed (*n* = 10). **(F, G)** Altered expression of MeJA signaling genes *OsMYC2* and *OsCOI1a* in detached leaves of 3-week-old WT and *oserf101* seedlings during DIS. Total RNA was isolated from detached leaves at 0, 2, 3, and 4 DDI as shown in **Figure 3A**. The transcript levels of *OsMYC2* **(F)** and *OsCOI1a* **(G)** were determined by qRT-PCR and normalized to that of *OsUBQ5* (Os01g22490). Mean and standard deviations were obtained from at least three biological samples. Different letters indicate significantly different values according to a one-way ANOVA and Duncan’s least significant range test (*p* < 0.05). FW, fresh weight.

In the WT, the expression of *OsERF101* increased in response to treatment with ABA or MeJA (**Figure 1B**), implying that *OsERF101* is positively involved in ABA- or JA-mediated leaf senescence. To assess the function of OsERF101 in JA signaling, we observed the progress of leaf yellowing in the detached leaves of 3-week-old WT and *oserf101* seedlings incubated in 3 mM MES buffer (pH 5.8) containing 50 µM of ABA or MeJA under continuous light conditions. While there was no significant difference in leaf greenness and chlorophyll contents between the WT and *oserf101* seedlings at 4 d of ABA treatment, the *oserf101* leaves retained their green color more than WT leaves at 4 d of MeJA treatment and had higher total chlorophyll contents (**Figures 4A, B**). Consistent with these observations, chlorophyll contents were higher in the whole leaves of 3-week-old *oserf101* seedlings than in those of the WT at 4 d of MeJA treatment (**Supplementary Figure S2**). In addition, we investigated the effects of MeJA on growth of WT and *oserf101* seedlings grown on 1/2 MS solid medium for 10 days. MeJA treatment significantly retarded the growth of shoots and roots in WT and *oserf101* seedlings (**Figure 4C**). However, the shoots and roots of the *oserf101* mutant were much longer than those of the WT under MeJA treatment (**Figures 4D, E**). These results suggested that, compared with WT, the *oserf101* mutant is less sensitive to MeJA.

To identify the effect of *OsERF101* on JA signaling and biosynthesis during DIS, we investigated the transcript levels of JA related genes in the detached leaves of 3-week-old WT and *oserf101* seedlings during DIS (**Figure 3A**). The expression of JA signaling genes (*OsMYC2* and *OsCOI1a*) and JA biosynthesis genes (*OsLOX2* and *OsAOS1*) was significantly upregulated in WT after 3 DDI, while the transcript levels were not altered in *oserf101* mutant during DIS (**Figures 4F, G** and **Supplementary Figure S3**). Taken together, our results suggested that promotes the onset and progression of leaf senescence by upregulating JA signaling and biosynthesis pathways.

### OsERF101 Binds to the *OsNAP* and *OsMYC2* Promoter Regions

**Figure 5:**
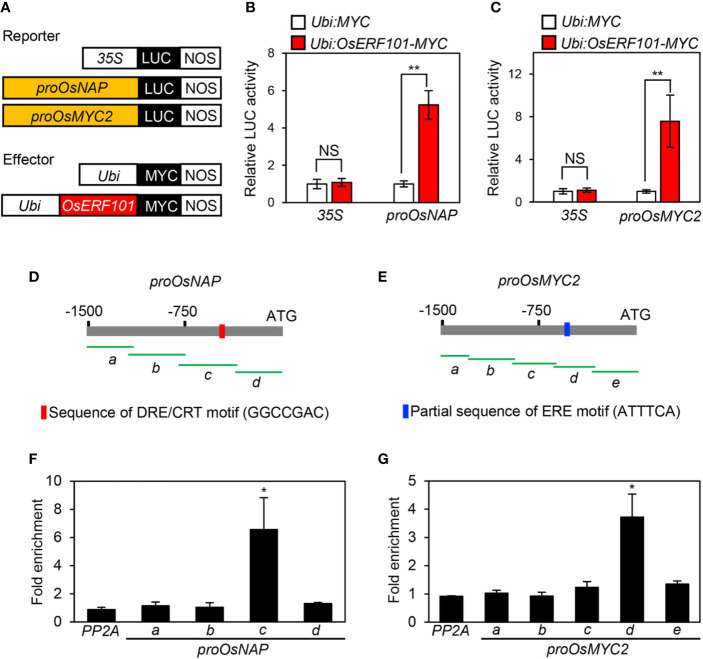
OsERF101 directly activates the transcription of *OsNAP* and *OsMYC2*. **(A)** Reporter and effector constructs used in the transactivation assay. **(B, C)** The activation of the *OsNAP* and *OsMYC2* promoter (*proOsNAP* and *proOsMYC2*) by OsERF101-MYC in the protoplast transient assay. The *35S* promoter was used as a negative control. **(D, E)** Positions of fragments used for the ChIP assay (green horizontal lines) in the promoter regions of *OsNAP* and *OsMYC2*. Red and blue bars indicate the position of the DRE/CRT and partial ERE binding motifs, respectively. **(F, G)** OsERF101 binding affinity assays to the promoter regions of *OsNAP* and *OsMYC2 in planta* examined by ChIP assays. OsERF101-Myc was transiently expressed in protoplasts isolated from 10-day-old WT seedlings. Fold-enrichment of the promoter fragments was measured by immunoprecipitation with an anti-Myc antibody (see *Methods*). The *SERINE/THREONINE PROTEIN PHOSPHATASE 2A* (*PP2A*) gene was used as a negative control. Mean and standard deviations were obtained from more than five biological repeats. Asterisks indicate a significant difference compared with the negative control (Student’s *t*-test, *p < 0.05, **p < 0.01).

During DIS, the expression of *OsNAP* and *OsMYC2* was downregulated in the *oserf101* mutant compared to the WT (**Figures 3E** and **4F**). Therefore, we used protoplast transient transactivation assays to determine if OsERF101 activates the transcription of *OsNAP* and *OsMYC2 in planta*. For the reporter construct, the promoter region of *OsNAP* (-1502 to -1) or *OsMYC2* (-1529 to -1) was fused with the luciferase (LUC) reporter (**Figure 5A**). The LUC activities of the protoplasts transformed with the *proOsNAP : LUC* and *proOsMYC2:LUC* plasmids were significantly enhanced when each of them was co-transfected with the *35S:OsERF101:MYC* effector plasmid (**Figures 5B, C**).

We conducted chromatin immunoprecipitation (ChIP) assays to examine whether OsERF101 directly binds to the promoter regions of *OsNAP* and/or *OsMYC2*. The 1500-bp region upstream of the transcription start site of *OsNAP* or *OsMYC2* was divided into four or five fragments with 50-bp overlap (**Figures 5D, E**). OsERF101 strongly bound to the amplicon-c region of *proOsNAP*, which includes the DRE/CRT motif (GGCCGAC, -447 to -440), and to the amplicon-d region of *proOsMYC2*, which includes a partial ERE motif (ATTTCA, -483 to -477) (**Figures 5F, G**). We further checked the expression levels of *OsNAP* and *OsMYC2* in the developing leaves of WT and *OsERF101*-OX plants grown for one month in the paddy field under NLD conditions. The qRT-PCR analysis revealed that transcripts of *OsNAP* and *OsMYC2* were significantly abundant in two *OsERF101*-OX lines than the transcript levels in the WT (**Supplementary Figure S4**). These results demonstrated that *OsERF101* functions as an upstream activator of *OsNAP* and *OsMYC2* by directly binding to their promoter regions.

### Loss of Function of *OsERF101* Negatively Affects Panicle Development and Grain Yield

**Figure 6:**
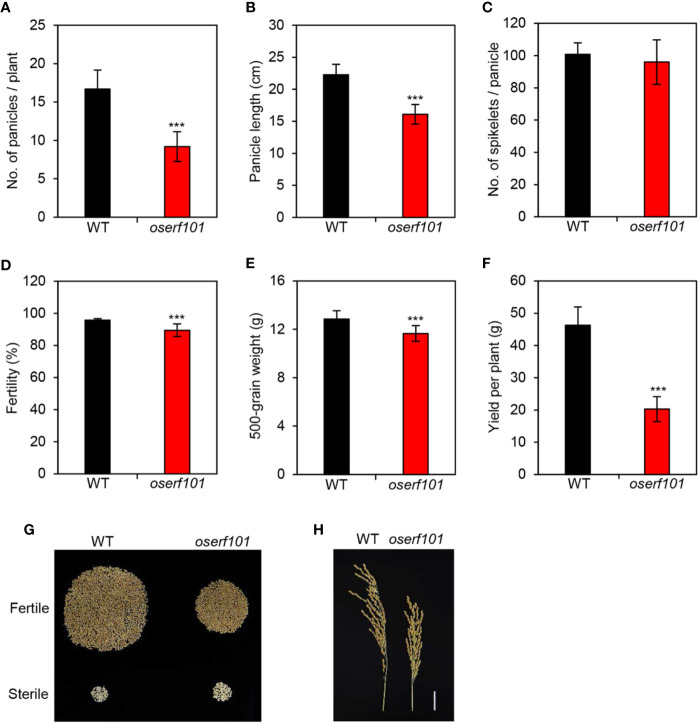
Agronomic traits measured in the *oserf101* mutant. Agronomic traits were compared between the WT and *oserf101* plants after harvest in the autumn. **(A)** Number of panicles per plant. **(B)** Panicle length. **(C)** Number of spikelets per panicle. **(D)** Fertility. **(E)** 500-grain weight. **(F)** Grain yield per plant. **(G)** The relative abundances of fertile and sterile spikelets in the WT and *oserf101* plants. **(H)** Phenotype of panicles. Mean and standard deviations were obtained from 10 rice plants. Asterisks indicate a significant difference between the WT and *oserf101* mutants (Student’s *t*-test, ***p < 0.001).

In addition to exploring the molecular genetic function of *OsERF101* in leaf senescence, we examined several agronomic traits in the *oserf101* mutant plants grown in the paddy field under NLD conditions. We evaluated the number of panicles per plant, panicle length, number of grains per panicle, fertility, and 500-grain weight. Interestingly, although the panicle length was shorter in the *oserf101* mutant than in the WT (**Figure 6A**), WT and *oserf101* plants produced a similar number of spikelets per panicle (**Figure 6B**), implying that the mutation of *OsERF101* led to higher spikelet density (**Figure 6H**). However, the number of panicles per plant, spikelet fertility, and 500-grain weight were lower in the *oserf101* mutant (**Figures 6C–E**), resulting in reduced grain yield per plant in the mutant (**Figures 6F, G**). Thus, the defect of panicle development and low spikelet fertility in the *oserf101* mutant appear to attenuate the advantage of high spikelet density on improving grain yield.

## Discussion

Leaf senescence largely occurs in an age-dependent manner, but can also be triggered by abiotic stresses. The onset and progression of leaf senescence are controlled by multiple regulatory networks in response to environmental cues. Leaf senescence is accompanied by chlorophyll degradation through phytohormone signaling pathways that are driven by many plant-specific TFs (Buchanan-Wollaston, 1997; Lee et al., 2001; Kim et al., 2016). *OsERF101* transcript levels were upregulated by treatment with phytohormones (ABA, MeJA), and by salt (NaCl), osmotic (PEG), and dehydration stresses (**Figures 1B–D**), implying that *OsERF101* is involved in senescence and abiotic stress responses through mediating ABA and MeJA signaling. Overexpression of *OsERF101* confers enhanced tolerance to osmotic and drought stresses at vegetative and reproductive stages, respectively (Jin et al., 2018). The finding that ABA-responsive genes such as *LATE EMBRYOGENESIS ABUNDANT 3* (*LEA3*), *PEROXIDASE 2* (*POD2*), and *RESPONSIVE TO DEHYDRATION 22* (*RD22*) are upregulated in *OsERF101*-overexpressed transgenic rice seedlings in response to osmotic stress treatment (20% PEG) suggesting that *OsERF101* participates in the ABA signaling pathway under osmotic stress conditions (Jin et al., 2018). In the present study, we found that overexpression of *OsERF101* led to early leaf yellowing during DIS and natural senescence (**Figure 3**). The detached leaves of the *oserf101* mutant were less sensitive to MeJA, but were sensitive to ABA in continuous light (**Figure 4** and **Supplementary Figure S2**), indicating that OsERF101 mediates leaf senescence *via* the JA signaling pathway, but not the ABA signaling pathway. Indeed, the expression of the JA signaling-associated genes *OsCOI1b* and *OsMYC2* was suppressed in the *oserf101* mutant during DIS (**Figures 4F, G**). Moreover, the ChIP assay showed that OsERF101 directly binds to a partial ERF motif in the promoter region of *OsMYC2* (**Figures 5E, G**). OsMYC2 directly regulates the expression of senescence-associated genes (*SIMILAR TO SAG* and *OsSAG12*), leading to rapid leaf yellowing in *OsMYC2*-OX plants (Uji et al., 2017). Together, these results support the idea that *OsERF101* acts as a positive regulator of leaf senescence in response to JA.

**Figure 7:**
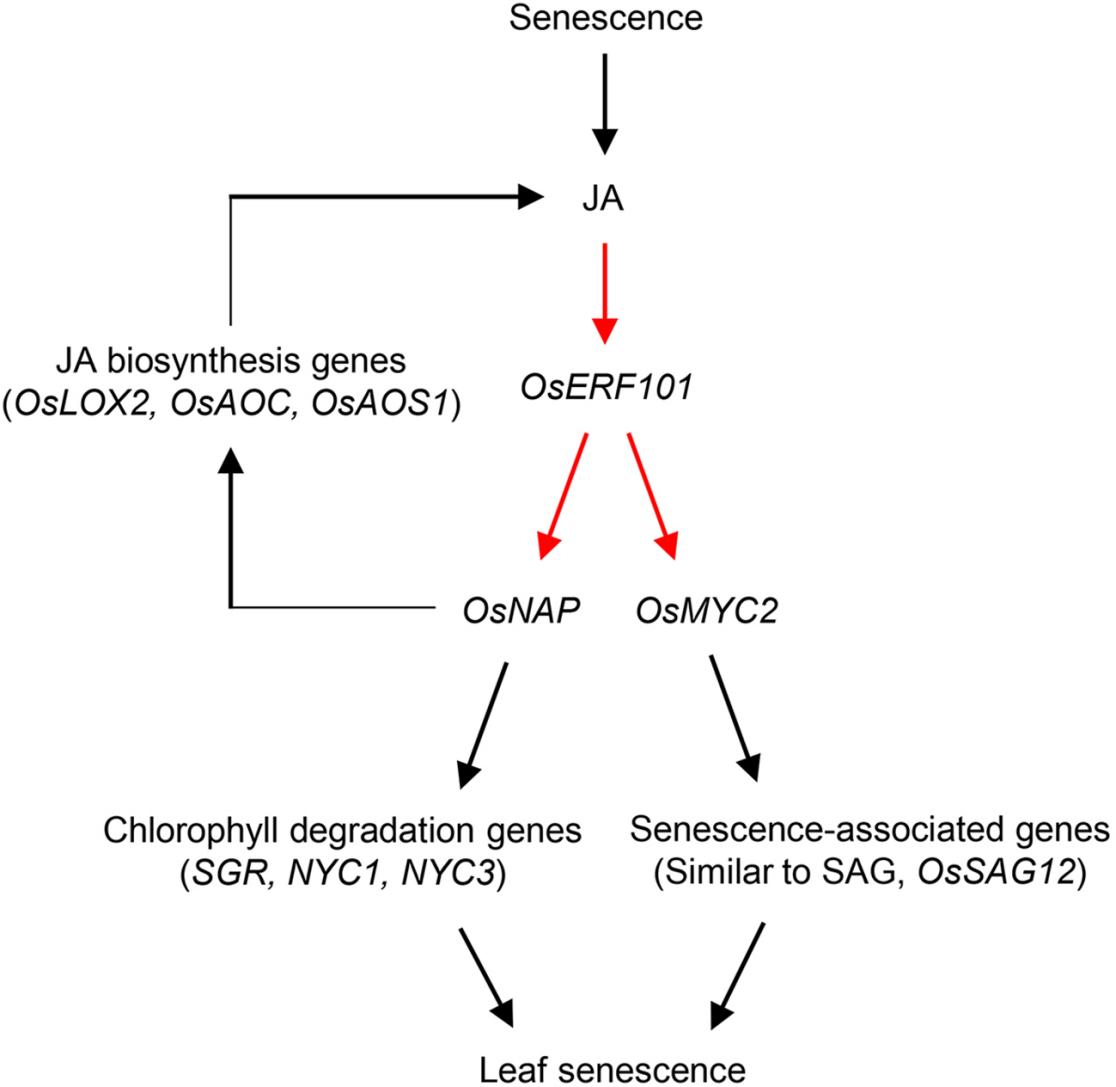
Proposed model of the role of *OsERF101* in the regulation in leaf senescence through JA signaling pathways involving *OsNAP* and *OsMYC2*. Red arrows indicate our findings in this study, and black arrows represent pathways identified in previous studies (Zhou et al., 2013; Liang et al., 2014; Uji et al., 2017).

In addition to the involvement of *OsERF101* in regulation of leaf senescence *via* the JA signaling pathway, *OsERF101* is involved in two independent senescence pathways mediated by OsNAP (**Figure 7**). The first is the *OsNAP*-mediated positive feedback loop modulating JA biosynthesis. OsERF101 activates *OsNAP* transcription by directly binding to the DRE/CRT motif of the *OsNAP* promoter region (**Figures 5D, F**). *OsNAP* is involved in various regulatory pathways regulating abiotic stress responses and leaf senescence. Here, we discussed the regulatory function of OsNAP in JA-regulated leaf senescence. Overexpression of *OsNAP* led to an increased level of expression of JA biosynthesis genes including *OsLOX2* and *OsAOC*, thereby upregulating endogenous JA levels in the transgenic plants (Zhou et al., 2013). Thus, the downregulation of JA biosynthesis genes (*OsLOX2* and *OsAOS1*) in the *oserf101* mutant during DIS suggests that *OsERF101* participates in JA biosynthesis (**Supplementary Figure S3**). Finally, increased levels of JA can enhance the transcription of *OsERF101*, and consequently activate the JA–OsERF101–OsNAP positive feedback regulatory loop to promote leaf senescence. The second regulatory function of OsNAP is the *OsNAP*-dependent chlorophyll degradation pathway. In addition, OsNAP is required for the induction of chlorophyll degradation genes including *SGR*, *NYC1*, and *NYC3* (Liang et al., 2014). Our observations indicated that the *oserf101* leaves retained their green color much longer than the WT due to the lower expression of *OsNAP* and chlorophyll degradation genes during DIS, indicating that *OsERF101* upregulates *OsNAP*-induced chlorophyll degradation.

In higher plants, endogenous JA levels and aqrJA signal transduction play important roles in shoot growth, lateral and adventitious root formation, seed germination, and embryo and pollen development (Ueda and Kato, 1980; McConn et al., 1997; Kang et al., 2010; Dave et al., 2011; Valenzuela et al., 2016). For instance, inhibition of JA signaling by knockdown of *OsCOI1a* and *OsCOI1b* expression led to increases in plant height, internode length, and cell length (Yang et al., 2012). Mutation of *OsMADS1* causes a defect in spikelet development due to a lack of glume formation (Chen et al., 2006). OsJAZ1 interacts with the OsMYC2 TF to suppress its activity, which leads to downregulation of the expression of *OsMADS1* (Cai et al., 2014). Genetic studies have indicated that the gain-of-function mutant of *OsJAZ1/EG2*, *eg2-1D*, exhibits downregulated expression of *OsMADS1*, resulting in low spikelet fertility. Thus, the poor spikelet fertility of the *oserf101* mutant appears to be caused by inhibition of JA signaling due to downregulation of *OsCOI1b* and *OsMYC2*, ultimately leading to lower grain yield (**Figures 6D, F**).

Taken together, we concluded that *OsERF101* affects multiple aspects of plant development, including leaf senescence and grain yield through regulating *OsNAP* and JA signaling. These results provide insight into the multiple regulatory mechanisms of *OsERF101* in leaf senescence and spikelet development in rice.

## Data Availability Statement

All datasets presented in this study are included in the article/**Supplementary Material**.

## Author Contributions

KK and N-CP designed experiments. CL, YSh, and YSa performed experiments and analyzed data. GA developed plant materials. CL, KK, YSh, and N-CP wrote the article.

## Funding

This work was carried out with the support of the Basic Science Research Program through the National Research Foundation (NRF) of Korea funded by the Ministry of Education (NRF-2017R1A2B3003310 to N-CP and NRF-2019R1I1A1A01060494 to KK).

## Conflict of Interest

The authors declare that the research was conducted in the absence of any commercial or financial relationships that could be construed as a potential conflict of interest.
