# Supplementary material for: Rice ETHYLENE RESPONSE FACTOR 101 Promotes Leaf Senescence Through Jasmonic Acid-Mediated Regulation of OsNAP and OsMYC2
Source: Front Plant Sci. 2020 Jul 16;11:1096. doi: 10.3389/fpls.2020.01096 (PMC7378735; doi:10.3389/fpls.2020.01096)
Supplement: Supplementary file 1 [file DataSheet_1.pdf]

SUPPLEMENTARY MATERIALS - Lim et al.

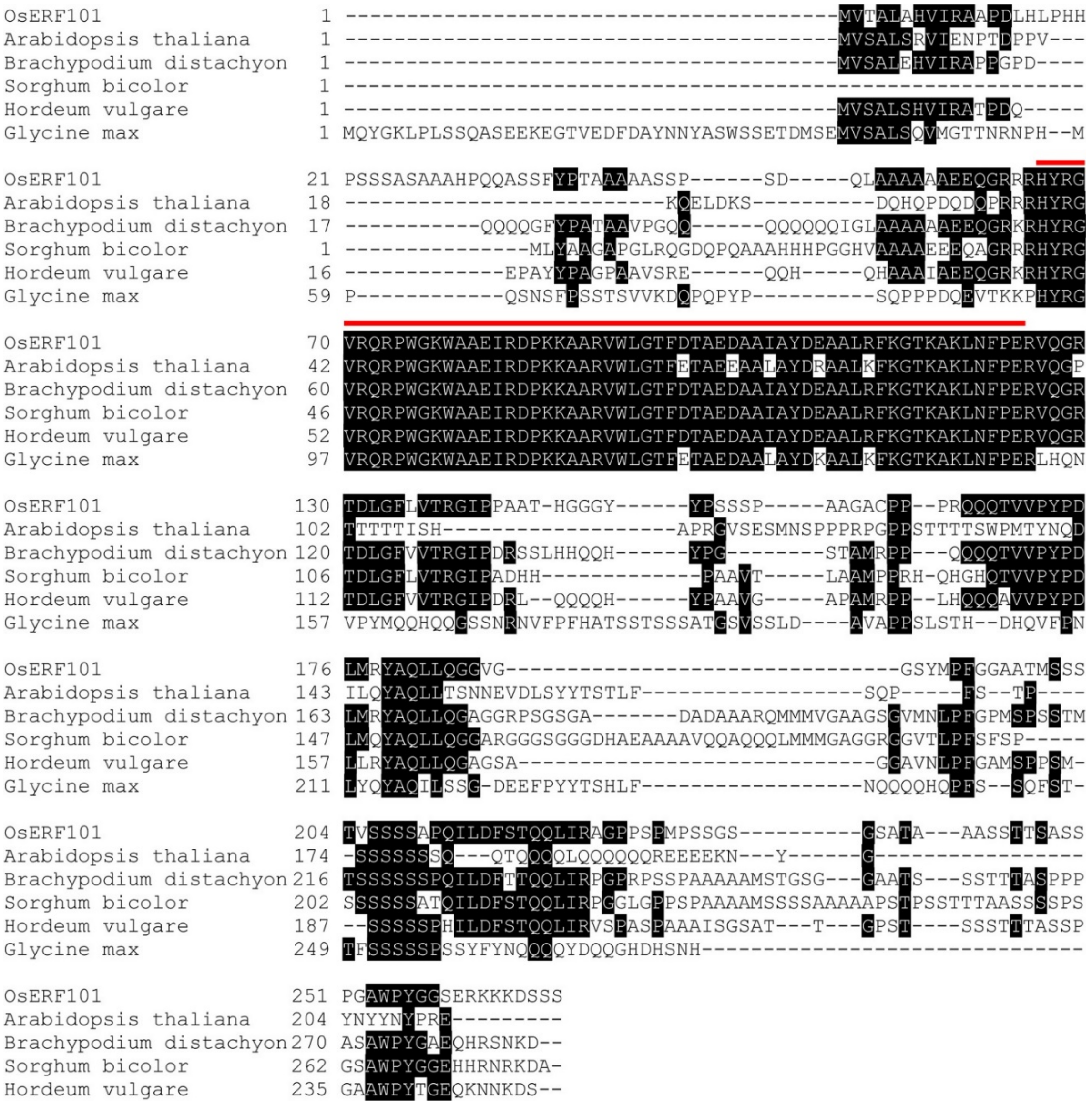

SUPPLEMENTARY FIGURE S1. Amino acid sequence alignment of ERF proteins.

The amino acid sequences of ERF proteins from rice and other plant species homologous to OsERF101 were obtained from NCBI through BLAST analysis. Sequence alignment was performed using ClustalW with default parameters. The sequences are as follows: *Oryza sativa*, XP_015636603.1; *Arabidopsis thaliana*, NP_196837.1; *Brachypodium distachyon*, XP_010239814.1; *Sorghum bicolor*, XP_021318101.1; *Hordeum vulgare*, BAJ99457.1; *Glycine max*, XP_003530479.2; The black boxes represent the conserved AP2/ERF domains of ERF proteins. The red bar represents the AP2/ERF domain of ERF proteins.

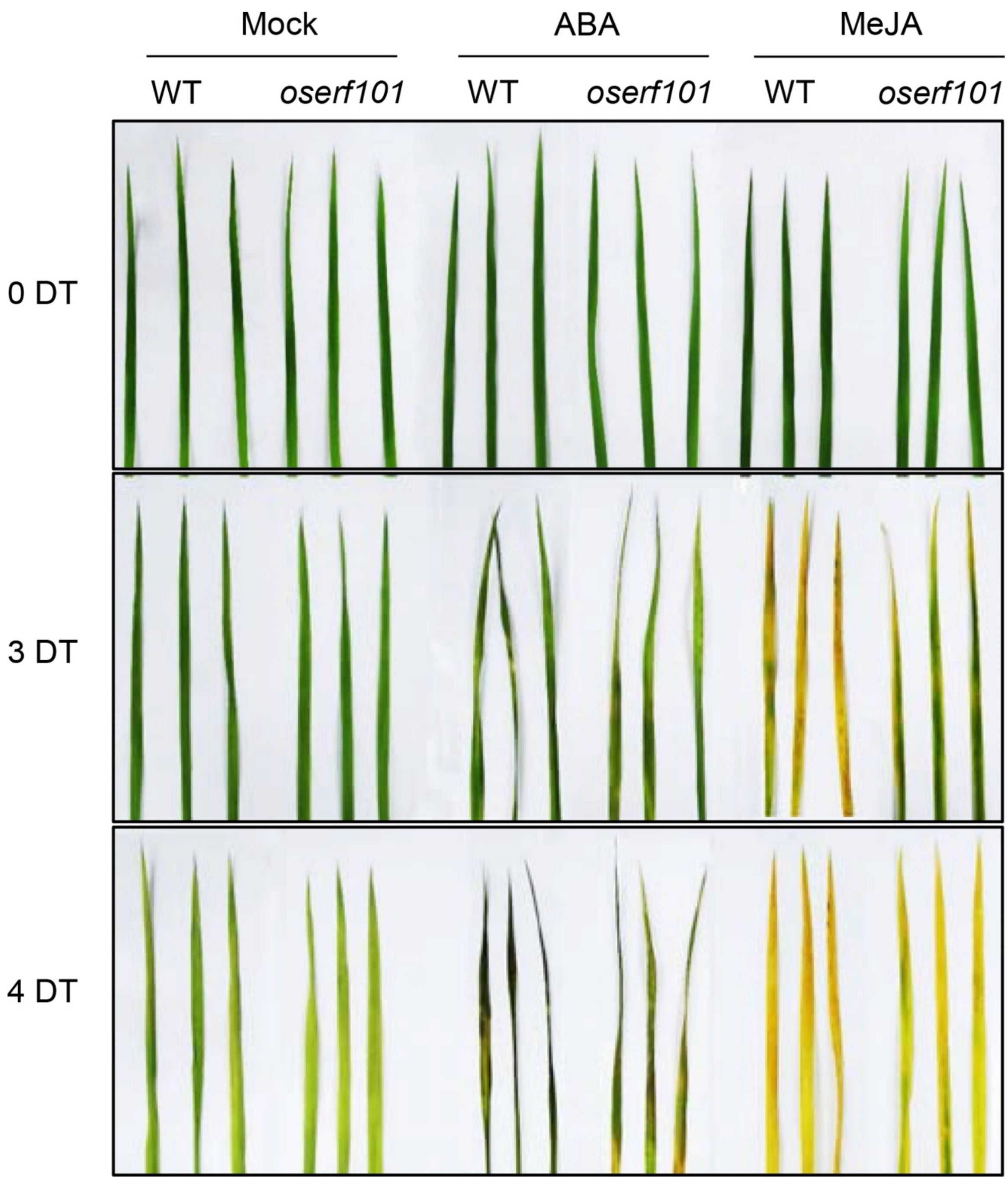

SUPPLEMENTARY FIGURE S2. The *oserf101* mutant is less sensitive to MeJA than the WT. Whole leaves of WT and *oserf101* plants grown in paddy soil for three weeks were incubated in 3 mM MES buffer (pH 5.8) containing 50 μM ABA or 50 μM MeJA under continuous light conditions at 28°C. Whole leaves floated on 3 mM MES buffer (pH 5.8) without phytohormones were used as a mock control. The pictures were taken at 0, 3, and 4 days after treatment (DT).

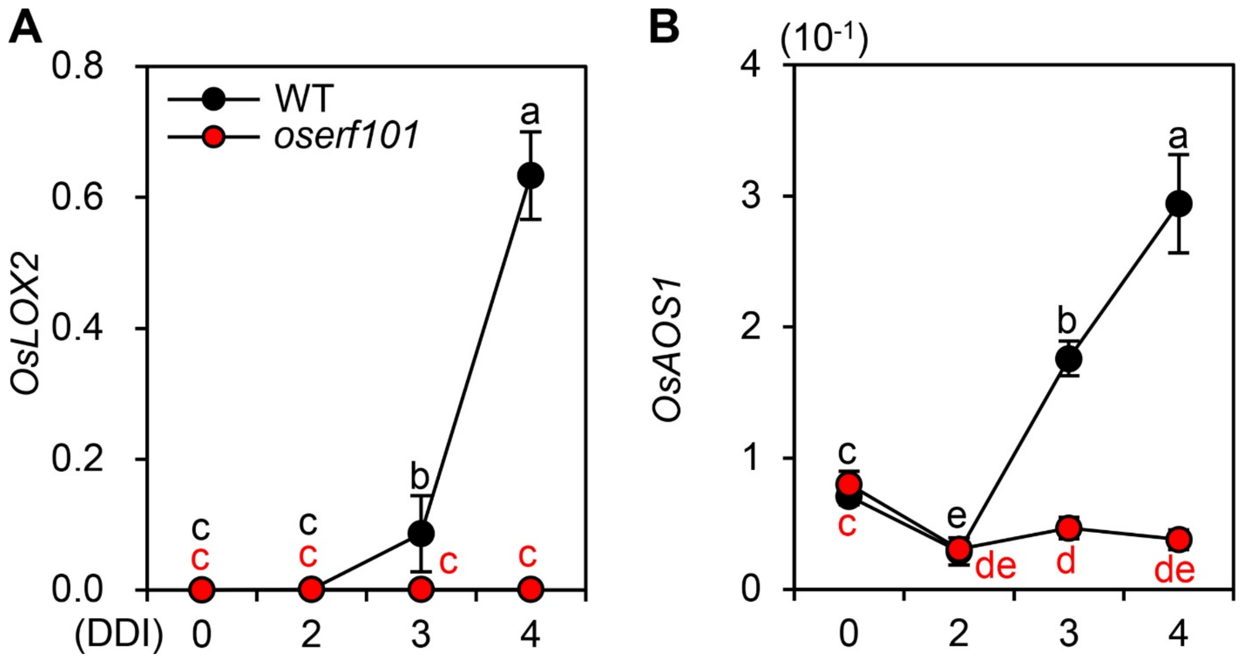

SUPPLEMENTARY FIGURE S3. Expression profiles of JA biosynthesis genes in the *oserf101* mutant during DIS. Total RNA was isolated from detached leaves at 0, 2, 3, and 4 days of dark incubation (DDI) as shown in **Figure 3A**. The transcript levels of *OsLOX2* **(A)** and *OsAOS1* **(B)** were determined by qRT-PCR and normalized to that of *OsUBQ5* (Os01g22490). Mean and standard deviations were obtained from three biological repeats. Different letters indicate significantly different values according to a one-way ANOVA and Duncan's least significant range test ($p < 0.05$).

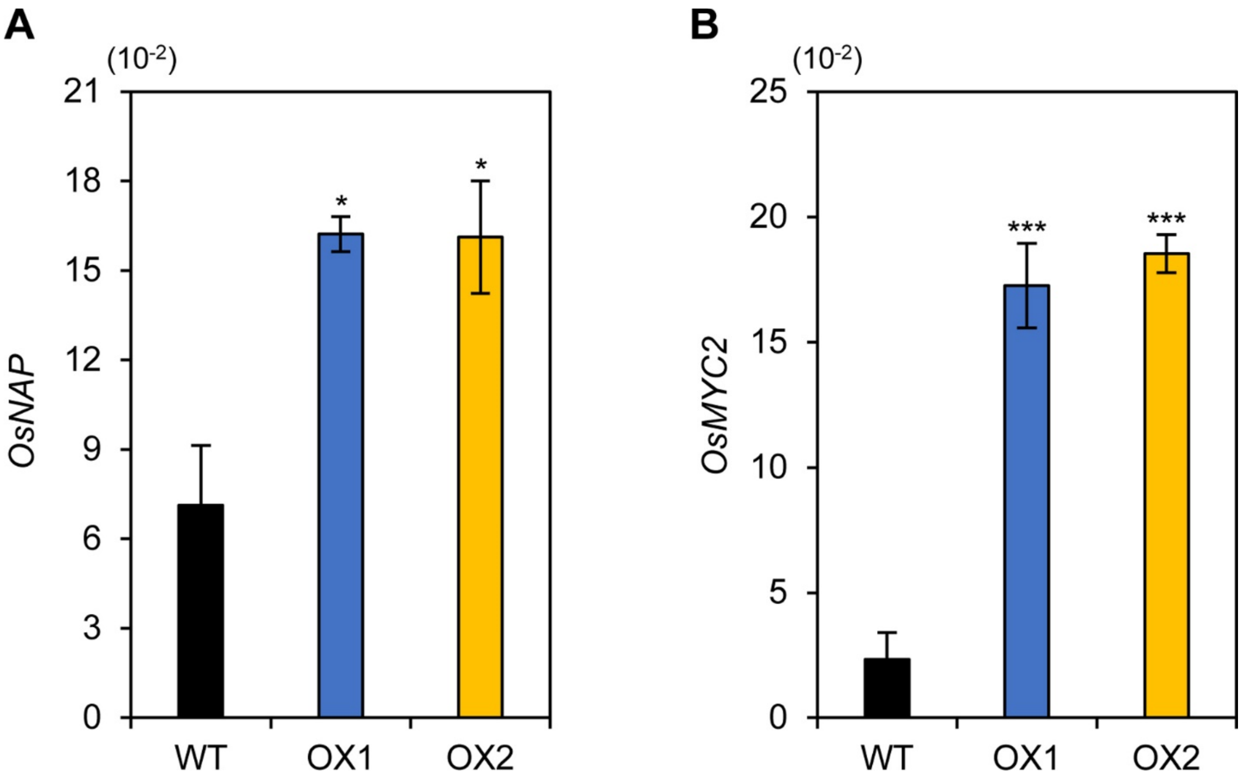

SUPPLEMENTARY FIGURE S4. Expression profiles of *OsNAP* and *OsMYC2* in the *OsERF101*-OX plants (OX1, OX2) grown for one month in the paddy field under NLD conditions. The transcript levels of *OsNAP* **(A)** and *OsMYC2* **(B)** were determined by qRT-PCR and normalized to that of *OsUBQ5* (Os01g22490). Mean and standard deviations were obtained from three biological repeats. Asterisks indicate statistically significant differences between treated samples and the mock control, as determined by Student's *t*-test (*$p < 0.05$, ***$p < 0.001$).

SUPPLEMENTARY TABLE S1. Primers used in this study.

	Forward primers (5'→3')	Reverse primers (5'→3')
A. primers for verification of transgenic plants		
PFG_2D-00368	TTGCATGCATTTTCATCTCC	TTAACTCCCAAATCGTTGCC
pGA2772	TCCGAAACTATCAGTGTCTAGCT	TTGGGGTTTCTACAGGACGTAAC
B. Primers used for gene cloning		
OsERF101	ATGGTCACCGCGCTAGCCCA	TCACGACGACGAATCCTTC
proNAP	AAACTGCAGAAACGTCTCATTCAGTAT	AAACCATGGGGCAGTCACCCA
proMYC2	AAACTGCAGGCCCTAACACAAATAC	AAACCATGGGGGTCCCATGGCTAA
C. Primers used for semi-quantitative RT-PCR		
OsERF101	ATGGTCACCGCGCTAGCCCA	TCACGACGACGAATCCTTC
OsUBQ5	ACCACTTCGACCGCCACTACT	ACGCCTAAGCCTGCTGGTT
D. Primers used for ChIP assays		
pOsNAP-a	GGAAACGTCTCATTCAGTATTAGGTTC	GGGCATTTTGGAAGTGTCAAGAATG
pOsNAP-b	CATTCTTGACACTTCCAAATGCCCTTA	GGTTACAGTGAACAGGAACACAGTTG
pOsNAP-c	CTGTGTTCCTGTTCACTGTAACCAAACTTC	GTGGGTTTGGTCCGTTATCCCT
pOsNAP-d	AAGCGCCGGAAACCACAGAAAA	GGCAGTCACCCACACACAACA
pOsMYC2-a	GCCCTAACACAAATACATTAAATGT	GATGATGCATACACCAAGTTTGTTAC
pOsMYC2-b	GTAACAAACTTGGTGTATGCATCATC	AAGAGTTACTCAAGCCTTAAGGCTAT
pOsMYC2-c	ATAGCCTTAAGGCTTGAGTAACTCTT	TAATCAGCCTCCGCTCTCGCTCGAT
pOsMYC2-d	ATCGAGCGAGAGCGGAGGCTGATTA	GTTTGCATGCATTTTGTATATCGTG
pOsMYC2-e	CACGATATACAAAATGCATGCAAAC	GGGTCCCATGGCTAATCACTCAAA
E. Primers used for RT-qPCR		
OsERF101	TGGTGCCATGTGTCGAGATT	GCACGGGATGGGGATGATAC
SGR	AGGGGTGGTACAACAAGCTG	GCTCCTTGCGGAAGATGTAG
NYC1	CATGCAACACCAACAAAAGG	GACCATTCCAGGAGAAGCAG
NYC3	TGTCGTTGCCATGTGAAGAT	TTGGTCACGCCACAAATCTA
OsNAP	CAAGAAGCCGAACGGTTC	GTTAGAGTGGAGCAGCAT
OsLOX2	GAGCCCGACTCCGGCAACCCTC	TCGCGTGGCACGTAGATGTTCTCC
OsAOS1	CAATACGTGTACTGGTCGAATGG	AAGGTGTCGTACCGGAGGAA
OsMYC2	CAGTGCCACAAGCGGAACCAC	GTTGAGCTGGTCCTGCGAGTAG
OsCOI1a	GATGCCCTCCCTGAGATACA	AGTCAGACCTCCTTCCAGCA
OsUBQ5	ACCACTTCGACCGCCACTACT	ACGCCTAAGCCTGCTGGTT
